# Caucasian validation of downstaging from IIB to IIA in T1N1M0 patients within the 9th edition of the non‐small cell lung cancer tumor‐node‐metastasis staging

**DOI:** 10.1002/cam4.70018

**Published:** 2024-07-24

**Authors:** Jingsheng Cai, Yun Li, Feng Yang

**Affiliations:** ^1^ Thoracic Oncology Institute and Research Unit of Intelligence Diagnosis and Treatment in Early Non‐small Cell Lung Cancer Peking University People's Hospital Beijing People's Republic of China; ^2^ Department of Thoracic Surgery Peking University People's Hospital Beijing People's Republic of China; ^3^ Institute of Advanced Clinical Medicine Peking University Beijing People's Republic of China

**Keywords:** downstaging, stage IIA, stage IIB, the 9th edition of the lung cancer TNM staging

## Abstract

**Background:**

The 9th edition of the lung cancer tumor‐node‐metastasis (TNM) staging introduced adjustments, including the reclassification of T1N1M0 patients from stage IIB to IIA. This update used data mostly from Asian populations. However, the applicability of these adjustments to Caucasian patients remains uncertain.

**Methods:**

Stage II non‐small cell lung cancer (NSCLC) patients from the Surveillance, Epidemiology, and End Results (SEER) database were included. Kaplan–Meier analysis with log‐rank testing compared overall survival (OS) and cancer‐specific survival (CSS). Propensity score matching (PSM) balanced baseline characteristics. The least absolute shrinkage and selection operator (LASSO)‐based Cox analyses identified prognostic factors.

**Results:**

Among 10,470 eligible stage II NSCLC patients (median age: 69 years; male: 53.1%), there were 2736 in stage IIA, 2112 in IIA New, and 5622 in IIB groups. Before PSM, survival outcomes of stage IIA New patients were similar to those of stage IIA patients but better than those of stage IIB. After PSM, stage IIA New and IIB patients showed similar survival rates (OS, *p* = 0.276; CSS, *p* = 0.565). Conversely, stage IIA New patients had worse outcomes than stage IIA patients (OS, *p* < 0.001; CSS, *p* = 0.005). LASSO‐based Cox analyses confirmed stage IIA New patients had inferior prognosis compared to stage IIA patients (OS HR: 1 vs. 1.325, *p* < 0.001; CSS HR: 1 vs. 1.327, *p* < 0.001).

**Conclusions:**

The downstaging of T1N1M0 patients from stage IIB to IIA in the 9th edition TNM staging remains unverified in Caucasians. Caution is warranted in assessing the staging and prognosis of these individuals. Further validation of our findings is necessary.

## Abstract 2

The downstaging of T1N1M0 patients from stage IIB to IIA in the 9th edition TNM staging requires validation in the Caucasian population. Utilizing data from the American SEER database, our study revealed that the prognosis of downstaged patients (stage IIA New) resembled that of stage IIB patients but was inferior to that of stage IIA patients. Herein, we proposed that further investigations are warranted to ascertain the optimal staging for the IIA New subgroup.

## 1 INTRODUCTION

The 9th edition of the lung cancer tumor‐node‐metastasis (TNM) staging, announced at the 2023 World Conference on Lung Cancer, introduced several adjustments to the overall staging, including the reclassification of T1N1M0 patients from stage IIB to IIA.^1^ Compared to the databases utilized in the 8th edition,^2^ the database for the 9th edition^3^ includes a total of 124,581 patients, with 87,043 deemed valid. Notably, there has been an increase in the proportion of Asian patients, rising from 44.0% in the 8th edition to 51.4% in the 9th edition.^3^

The new version of the lung cancer TNM staging requires validation from external datasets. There are documented differences in survival rates, prognostic factors, and genetic molecular characteristics between Caucasian and Asian lung cancer populations.^4^, ^5^, ^6^ Whether the modifications are applicable to the Caucasian population remains to be further explored. Given the great significance of the lung cancer TNM staging system in clinical practice, it is imperative to further validate and explore the staging adjustments using data from Caucasian populations.

Based on the above, this study included resected stage II NSCLC patients from the American Surveillance, Epidemiology, and End Results (SEER) database. According to the 9th edition of the lung cancer TNM staging system, stage II patients were classified into IIA, IIA NEW (patients downstaged from IIB to IIA), and IIB groups. We aimed to explore whether there are prognostic differences among these three groups to elucidate the rationale behind the staging adjustment in the Caucasian population.

## 2 METHODS

### 2.1 Study population

**FIGURE 1:**
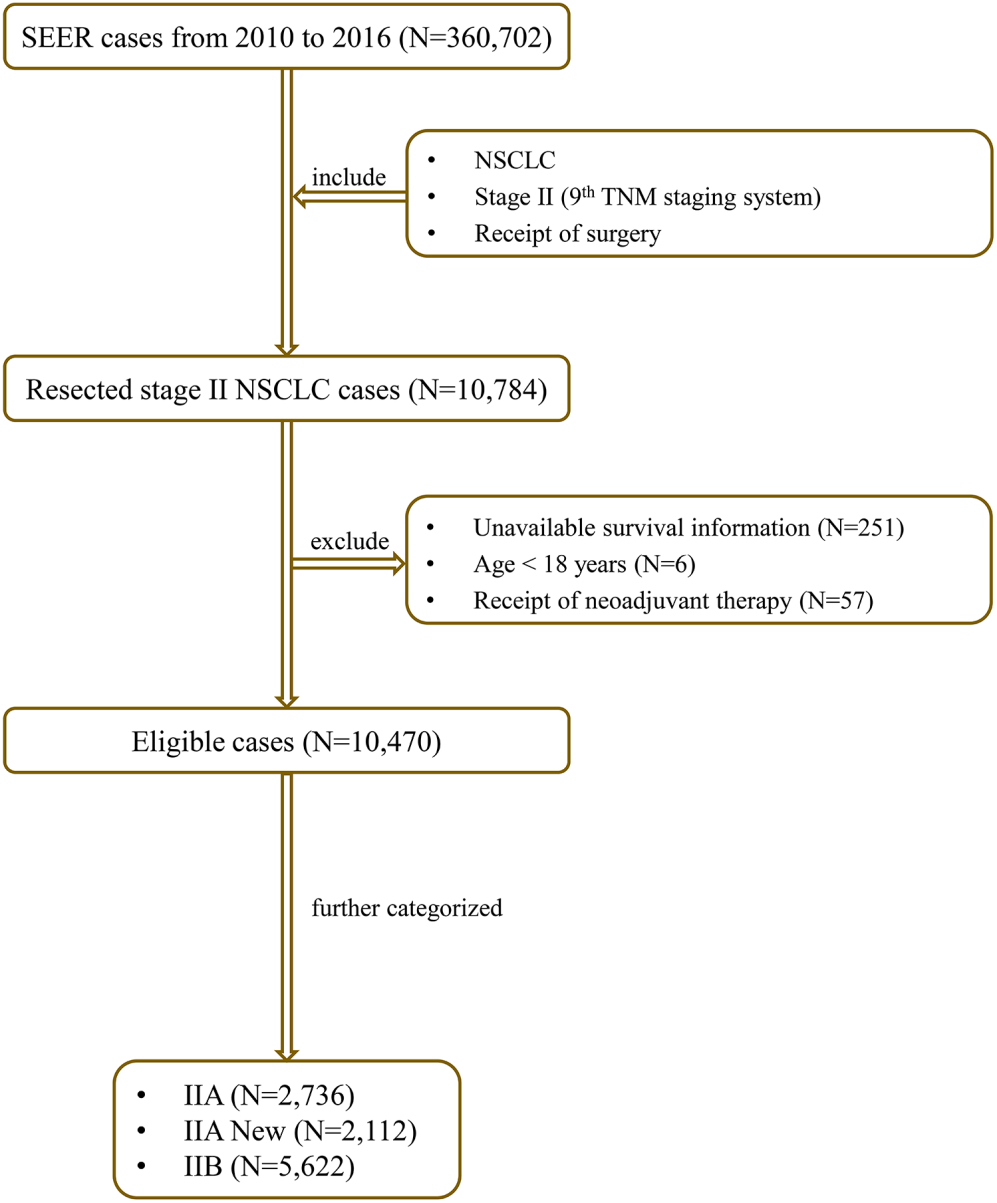
Patient selection flowchart. NSCLC, non‐small cell lung cancer; SEER, the Surveillance, Epidemiology, and End Results database; TNM, tumor‐node‐metastasis; IIA New, patients downstaged from IIB to IIA.

Lung malignancy cases from 2010 to 2016 were retrospectively reviewed from the SEER program (https://seer.cancer.gov). In this study, the inclusion criteria comprised: (1) diagnosis of NSCLC; (2) stage II (as per the 9th edition of TNM staging^1^); and (3) receipt of surgery. The exclusion criteria encompassed: (1) unavailable survival information and TNM staging; (2) Age under 18 years; and (3) receipt of neoadjuvant therapy. The eligible stage II cases were further categorized into three groups: IIA, IIA New (patients downstaged from IIB to IIA), and IIB. The exact patient selection flowchart is shown in Figure 1. In the 9th edition staging database, there were 19,608 cases from North America, comprising 15.7% of the total.^3^ Since we were unable to access the IASLC database, we are uncertain about which North American patients were included in their cohort. However, it is certain that our cohort included a much larger number of stage II cases compared to the IASLC database (the entire database contains only 7909 pathologically diagnosed stage II cases).^3^ Therefore, without a doubt, our cohort can be considered an external cohort.

### 2.2 Ethic

Given the retrospective nature and de‐identified data, individual informed consents were waived for this study. Access to the SEER data was granted under code 12962‐Nov2019. The study adhered to the principles outlined in the Helsinki Declaration of 1964 and its subsequent revisions.

### 2.3 Data collection

Patient data were retrieved from the electronic data sheet maintained in the SEER program, encompassing various epidemiological characteristics such as age, sex, race, insurance, marital status, survival status, survival time and cause of death. Additionally, tumor features including location, size, histology, grade, visceral pleura invasion (VPI), T category and N category, as well as treatment information such as surgical extent, radiotherapy and chemotherapy, were collected. The primary endpoints of this study were overall survival (OS) and cancer‐specific survival (CSS). OS was computed from the date of diagnosis to the date of death or last follow‐up. CSS was computed from the date of diagnosis to the date of death attributable to NSCLC or last follow‐up. Missing values for baseline covariates were handled as dummy variables, and patients lacking survival data were completely excluded.

### 2.4 Statistical analysis

SEER*Stat software version 8.3.4. (https://seer.cancer.gov/seerstat) facilitated data extraction from SEER program. Statistical analyses were conducted using IBM SPSS Statistics software version 25.0 (IBM Corp, Armonk, NY, USA) and R software version 4.1.1 (The R Foundation for Statistical Computing, Vienna, Austria; https://www.r‐project.org). Categorical variables were presented as number and percentage and compared using Chi‐squared test or Fisher's exact test. The normality of data distribution was assessed using the Shapiro–Wilk test. Non‐normally distributed continuous variables were presented as median and interquartile range (IQR) and compared using the Kruskal–Wallis *H* test and Mann–Whitney *U*‐test. Survival curves were plotted using the Kaplan–Meier method, with survival differences among groups evaluated using the log‐rank test. Bonferroni's adjustment was applied in multiple subgroup 1:1 analysis. One‐to‐one propensity score matching (PSM)^7^ was performed to mitigate bias arising from unbalanced baseline characteristics, utilizing the R package “MatchIt” (method = nearest, replace = FALSE). Standardized Mean Difference (SMD) was employed to assess covariate balance. A caliper distance of 0.00005 was used for both the IIA and IIA New pair and the IIA New and IIB pair. A least absolute shrinkage and selection operator (LASSO) regression model was utilized to select and minimize potential prognostic variables using the R package “glmnet.”^8^ The identified factors were subsequently included in a multivariable Cox analysis (forced enter method) to ascertain the final prognostic factors. Two‐sided *p*‐values < 0.05 were considered statistically significant.

## 3 RESULTS

### 3.1 Patient characteristics

From 2010 to 2016, a series of 360,702 lung malignancy cases were reviewed. Following the predefined inclusion and exclusion criteria, a total of 10,470 stage II cases were identified as eligible for analysis, comprising 2736 cases in the stage IIA group, 2112 in the stage IIA New group, and 5622 in the stage IIB group. The median age of the entire cohort was 69 years, with a relatively balanced gender distribution (male: 53.1%). Caucasians constituted the majority of the cohort (84.2%). The predominant surgical procedure performed was lobectomy (86.4%). Chemotherapy was administered to nearly half of the patients (43.6%), while radiotherapy was received by only a small minority (9.9%). The majority of patients were diagnosed with moderate/low‐grade tumors (81%). Baseline characteristics stratified by different TNM stagings are presented in Table 1. Apart from race (*p* = 0.749), marital status (*p* = 0.482), and insurance (*p* = 0.165), significant variances were observed among the three groups for other clinical‐pathological variables (all *p* < 0.001). It is noteworthy that the proportion of patients receiving chemotherapy is significantly higher in the IIA NEW and IIB groups compared to the IIA group (56.8% vs. 47.2% vs. 26.0%). Additionally, patients in the IIA NEW group have much smaller tumors compared to the other two groups (median: 21 mm vs. 45 mm vs. 48 mm).

**TABLE 1:** The Clinicopathological characteristic of stage II NSCLC patients.

Characteristic	IIA (*N* = 2735)	IIA new (*N* = 2112)	IIB (*N* = 5622)	*p*‐values
Age, years				
Continue (median, IQR)	70 (63–77)	67 (61–73)	68 (62–75)	<0.001^a^
Sex				
Male	1466 (53.6)	1027 (48.6)	3071 (54.6)	<0.001
Female	1270 (46.4)	1085 (51.4)	2551 (45.4)	
Race				
Caucasian	2322 (84.9)	1780 (84.3)	4711 (83.8)	0.749
African	239 (8.7)	194 (9.2)	517 (9.2)	
Other	175 (6.4)	138 (6.5)	394 (7.0)	
Marital status				
Married	1518 (55.5)	1199 (56.8)	3195 (56.8)	0.482
Other	1218 (44.5)	913 (43.2)	2427 (43.2)	
Insurance				
Insured	2388 (87.3)	1836 (86.9)	4828 (85.9)	0.165
Other	348 (12.7)	276 (13.1)	794 (14.1)	
Tumor location				
RUL	1504 (55.0)	1242 (58.8)	3089 (54.9)	<0.001
RML	129 (4.7)	138 (6.5)	270 (4.8)	
RLL	1006 (36.8)	663 (31.4)	2008 (35.7)	
LUL	45 (1.6)	57 (2.7)	136 (2.4)	
LLL	52 (1.9)	12 (0.6)	119 (2.1)	
Surgical extent				
Lobectomy	2413 (88.2)	1831 (86.7)	4797 (85.3)	<0.001
Sub‐lobectomy	221 (8.1)	172 (8.1)	345 (6.1)	
Pneumonectomy	102 (3.7)	109 (5.2)	480 (8.5)	
Chemotherapy				
Not performed	2025 (74.0)	912 (43.2)	2971 (52.8)	<0.001
Performed	711 (26.0)	1200 (56.8)	2651 (47.2)	
Radiotherapy				
Not performed	2557 (93.5)	1909 (90.4)	4964 (88.3)	<0.001
Performed	179 (6.5)	203 (9.6)	658 (11.7)	
Histology				
Adenocarcinoma	1142 (41.7)	1092 (51.7)	2275 (40.5)	<0.001
Squamous cell carcinoma	951 (34.8)	435 (20.6)	2003 (35.6)	
Other	643 (23.5)	585 (27.7)	1344 (23.9)	
Grade				
Well	370 (13.5)	201 (9.5)	470 (8.4)	<0.001
Moderately	1048 (38.3)	931 (44.1)	2194 (39.0)	
Poor	1053 (38.5)	780 (36.9)	2473 (44.0)	
Undifferentiated	57 (2.1)	31 (1.5)	138 (2.5)	
Unknown	208 (7.6)	169 (8.0)	347 (6.2)	
Tumor size, mm				
Continue (median, IQR)	45 (44–49)	21 (16–25)	48 (35–58.25)	<0.001^a^
VPI				
Without	2055 (75.1)	2112 (100.0)	3392 (60.3)	<0.001
With	681 (24.9)	0 (0.0)	2230 (39.7)	

Abbreviations: IQR, interquartile range; LLL, left low lobe; LUL, left upper lobe; NSCLC, non‐small cell lung cancer; RLL, right low lobe; RML, right middle lobe; RUL, right upper lobe; VPI, visceral pleural invasion.

^a^ Kruskal–Wallis *H* test.

After PSM, the IIA New and IIA matched group comprised 516 pairs, while the IIA New and IIB matched group consisted of 642 pairs. The baseline covariates between the matched groups were well balanced (Table S1).

### 3.2 Survival analysis

**FIGURE 2:**
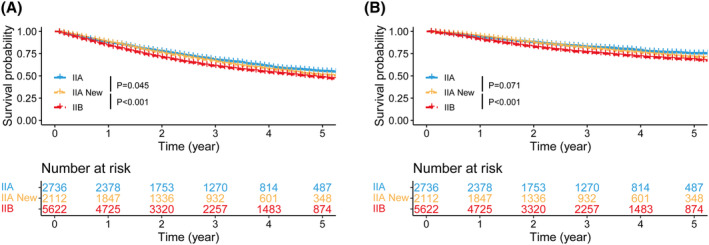
Survival comparisons among stage IIA, IIA New and IIB patients. (A) OS and (B) CSS. CSS, cancer‐specific survival; OS, overall survival; IIA New, patients downstaged from IIB to IIA.

The entire cohort of stage II patients was categorized into three groups: IIA, IIA New, and IIB, and their survival outcomes were compared. The data showed that patients in the IIA and IIA New group had similar OS (5‐year OS rate: IIA vs. IIA New = 55.1% vs. 50.7%, *p* = 0.045, the Bonferroni's adjustment requires that the significance level be set at *p* < 0.05/3 to maintain significance, Figure 2A) and CSS (5‐year CSS rate: IIA vs. IIA New = 75.1% vs. 71.0%, *p* = 0.071, Figure 2B). Additionally, the survivals of stage IIA New patients were significantly superior to those of stage IIB patients (5‐year OS rate: IIA New vs. IIB = 50.7% vs. 48.1%, *p* < 0.001, Figure 2A; 5‐year CSS rate: IIA New vs. IIB = 71.0% vs. 68.1%, *p* < 0.001, Figure 2B).

**FIGURE 3:**
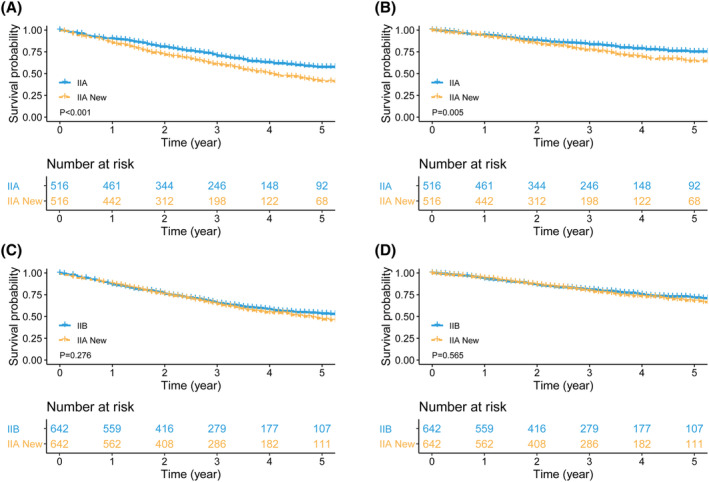
Survival comparisons between stage IIA New and IIA patients, and between stage IIA New and IIB patients after PSM. (A) OS: IIA New vs. IIA; (B) CSS: IIA New vs. IIA; (C) OS: IIA New vs. IIB; and (D) CSS: IIA New vs. IIB. PSM, propensity score matching; IIA New, patients downstaged from IIB to IIA.

Following PSM, survival analysis of the IIA New and IIA matched group indicated that the survivals of stage IIA New patients were inferior to those of stage IIA patients (5‐year OS rate: IIA New vs. IIA = 41.2% vs. 57.4%, *p* < 0.001, Figure 3A; 5‐year CSS rate: IIA New vs. IIA = 64.2% vs. 75.1%, *p* = 0.005, Figure 3B). In the IIA New and IIB matched group, comparable survival rates were observed between the two groups (5‐year OS rate: IIA New vs. IIB = 46.5% vs. 53.2%, *p* = 0.276, Figure 3C; 5‐year CSS rate: IIA New vs. IIB = 67.5% vs. 71.4%, *p* = 0.565, Figure 3D).

### 3.3 LASSO‐based multivariable Cox analysis

**FIGURE 4:**
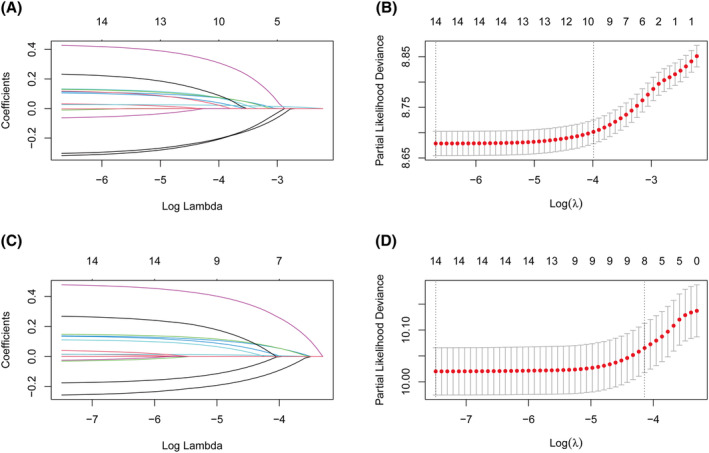
Selection of prognostic variables using LASSO model analysis. Profiles of LASSO coefficients for 14 covariates plotted against the log (Lambda) sequence for OS (A) and CSS (C). Tuning parameter (Lambda) selection in the LASSO model was determined using 10‐fold cross‐validation based on minimum criteria for OS (B) and CSS (D); CSS, cancer‐specific survival; LASSO, least absolute shrinkage and selection operator; OS, overall survival.

Clinical‐pathological variables, encompassing age, sex, race, marital status, insurance, tumor location, surgical extent, chemotherapy, radiotherapy, histology, tumor grade, tumor size, stage II category, and VPI, were incorporated into the LASSO model for both OS (Figure 4A) and CSS (Figure 4C) analyses. The lambda.1se criterion was employed to establish the most concise model within a variance range centered around lambda.min. Ultimately, the LASSO model for OS identified 10 variables, including age, sex, marital status, insurance, surgical extent, chemotherapy, radiotherapy, tumor grade, stage II category, and VPI (Figure 4B). Meanwhile, the LASSO model for CSS identified 8 variables, including age, sex, insurance, chemotherapy, radiotherapy, tumor grade, stage II category, and VPI (Figure 4D).

Following LASSO selection, the chosen variables were incorporated into the multivariable Cox regression models. The results demonstrated that stage II category was a significant prognostic factor for both OS (HR: IIA vs. IIA New vs. IIB = 1 vs. 1.325 vs. 1.294, *p* < 0.001, Table 2) and CSS (HR: IIA vs. IIA New vs. IIB = 1 vs. 1.327 vs. 1.296, *p* < 0.001, Table 2).

**TABLE 2:** Prognosis analysis: LASSO‐based multivariable Cox models for stage II NSCLC patients.

Characteristic	OS			CSS		
	HR	95% CI	*p*‐value	HR	95% CI	*p*‐value
Age, years						
Continue	1.026	1.023–1.030	<0.001	1.026	1.023–1.030	<0.001
Sex						
Male	1		<0.001	1		<0.001
Female	0.738	0.692–0.786		0.756	0.711–0.804	
Marital status						
Married	1		<0.001			
Other	1.131	1.062–1.205				
Insurance						
Insured	1		<0.001	1		<0.001
Other	1.245	1.139–1.362		1.289	1.180–1.409	
Surgical extent						
Lobectomy	1		<0.001			
Sub‐lobectomy	1.239	1.110–1.382				
Pneumonectomy	1.297	1.152–1.460				
Chemotherapy						
Not performed	1		<0.001	1		<0.001
Performed	0.705	0.659–0.754		0.702	0.656–0.750	
Radiotherapy						
Not performed	1		<0.001	1		<0.001
Performed	1.527	1.393–1.674		1.549	1.414–1.697	
Grade						
Well	1		<0.001	1		<0.001
Moderately	1.619	1.421–1.845		1.625	1.426–1.851	
Poor	1.980	1.740–2.253		1.995	1.753–2.270	
Undifferentiated	2.243	1.786–2.815		2.302	1.834–2.890	
Unknown	1.583	1.333–1.880		1.610	1.356–1.912	
VPI						
Without	1		0.002			
With	1.121	1.044–1.203				
Stage II						
IIA	1		<0.001	1		<0.001
IIA New	1.325	1.204–1.459		1.327	1.205–1.461	
IIB	1.294	1.199–1.396		1.296	1.202–1.398	

Abbreviations: CI, confidence interval; CSS, cancer‐specific survival; HR, hazard ratio; LASSO, least absolute shrinkage and selection operator; NSCLC, non‐small cell lung cancer; OS, overall survival; VPI, visceral pleural invasion.

## 4 DISCUSSION

Considering that the majority of the reference population included in the 9th edition staging comprises Asian individuals, with a limited representation of white individuals, external validation of the new TNM staging adjustments is imperative within the white population. This study focused on the inclusion of Caucasian patients with stage II NSCLC, aiming to explore whether the adjustments made to the 9th edition staging could be externally validated within this specific ethnic group. After rigorous patient inclusion/exclusion criteria and statistical analysis, our results indicated that the prognosis of downstaged patients (IIA New) was similar to that of stage IIB patients but inferior to that of stage IIA patients. Hence, we proposed that further investigations are warranted to ascertain the optimal staging for the IIA New subgroup. Our conclusion was not contradictory to the 9th edition staging; rather, it offered insights that call for careful consideration in staging stage II cases.

In the 9th edition staging,^1^ stage IIA patients were defined as T2bN0M0, whereas our newly defined IIA New subgroup refereed to patients with T1N1M0 status. The 9th edition staging categorized both into stage IIA, whereas our findings indicated that these two subgroups should not be classified uniformly as stage IIA. Despite variations in age, chemotherapy administration rates and histology between the two cohorts, survival comparisons after PSM consistently indicated poorer outcomes for stage IIA New patients. In our view, the primary reason for this discrepancy stemmed from the presence of lymph node involvement in stage IIA New patients, signifying an advancement of the tumor beyond localized growth within a single lung lobe and the acquisition of specific invasive and metastatic properties. Therefore, the high invasiveness of the tumor is one potential factor contributing to the poorer prognosis observed in stage IIA New patients.

Recently, Wang et al. utilized the SEER database to validate the 9th edition staging system for postoperative stage I‐IIIA patients.^9^ Their survival analysis revealed that among the IIB subgroup classified by the 8th edition, patients with T1N1M0 had similar outcomes to the remaining IIB patients (T2N1M0 + T3N0M0).^9^ This finding aligns closely with our current results. In our study, we further employed PSM and LASSO‐based multivariable Cox methods to balance other confounding factors, confirming that the prognosis for the T1N1M0 (IIA NEW) cohort is indeed consistent with that of the IIB stage. While currently there are no apparent differences in treatments for stage IIA and IIB patients,^10^ categorizing T1N1M0 patients into these stages may seem less meaningful. However, precise TNM staging served as the cornerstone for guiding patient treatment throughout the entire course. In current clinical practice, when encountering patients with T1N1M0, clinicians typically diagnose them as stage IIA rather than IIB. However, our findings reveal that their prognostic outcomes resemble those of stage IIB patients. Based on the 8th edition staging,^11^ their actual 5‐year OS rate is approximately 7% lower than currently assumed. Although actual survival may be worse, the downstaging of these patients may lead clinicians to consider relatively less aggressive treatment strategies or adopt more relaxed follow‐up schedules compared to previous practices. This may potentially compromise the survival benefit for stage IIB New patients.

While researchers aim for the TNM staging system to include more influencing factors for precise prognosis assessment across stages and treatments, it is essential to heed Detterbeck's view that “staging isn't meant to encompass all prognostic and treatment factors. It's not a prognostic model or treatment guideline but serves as a tool for prognostic assessment and aiding treatment decisions.”^12^ Indeed, the TNM staging system serves as a framework to guide treatment decisions for physicians and patients. In today's era of targeted therapy and immunotherapy, integrating molecular biomarkers of lung cancer such as driver gene mutations, PD‐L1 expression levels, etc., with TNM staging can significantly enhance patient management and outcomes.

Our study still has certain limitations: first, we only included patients who underwent surgical resection, which may introduce some selection bias; however, this was necessary to ensure staging accuracy. Second, our study only explored the pathological stage II classification, and further validation is needed to confirm whether the findings apply to clinical staging. Thirdly, our analysis did not include the T1N2aM0 patients who were downstaged to stage IIB due to the paucity of detailed lymph node metastasis data in the SEER database. Consequently, the comparability in prognosis between IIA New and IIB patients necessitates additional validation. Lastly, being retrospective in nature, our study is subject to inherent biases. Therefore, larger sample sizes and more comprehensive databases are needed to validate our conclusions.

## 5 CONCLUSIONS

In conclusion, our findings suggested that patients reclassified to stage IIA (T1N1M0) in the 9th edition staging system (IIA New) exhibited a prognosis inferior to other stage IIA patients yet akin to stage IIB patients. This underscored the importance of a cautious approach in evaluating the staging and prognosis of these individuals. Nevertheless, our conclusions warranted further validations.

## AUTHOR CONTRIBUTIONS

**Jingsheng Cai:** Data curation (equal); formal analysis (equal); investigation (equal); methodology (equal); software (equal); writing – original draft (equal); writing – review and editing (equal). **Yun Li:** Project administration (equal); writing – original draft (supporting); writing – review and editing (supporting). **Feng Yang:** Conceptualization (equal); project administration (equal); supervision (equal); writing – original draft (equal); writing – review and editing (equal).

## FUNDING INFORMATION

This work was supported by Research Unit of Intelligence Diagnosis and Treatment in Early Non‐small Cell Lung Cancer, Chinese Academy of Medical Sciences (2021RU002), CAMS Innovation Fund for Medical Sciences (CIFMS, 2022‐I2M‐C&T‐B‐120) and the National Natural Science Foundation of China (Number: 92059203).

## CONFLICT OF INTEREST STATEMENT

The authors declare no conflict of interest.

## ETHICS STATEMENT

The study was approved by the Ethics Committee of Peking University People's Hospital and conducted in accordance with the Declaration of Helsinki. Authorization to access SEER data was obtained using reference code 12962‐Nov2019. As the study utilized de‐identified data from the SEER database, individual informed consent forms were waived.

## CLINICAL PRACTICE POINTS

1. The database for the 9th edition staging predominantly comprises Asian populations, highlighting the necessity to validate staging adjustments within the Caucasian population.
2. Patients reclassified to stage IIA (T1N1M0) in the 9th edition staging system (IIA New) exhibited a prognosis that was inferior to other stage IIA patients but similar to that of stage IIB patients.
3. The importance of a cautious approach in assessing the staging and prognosis of these downstaged individuals (T1N1M0).

## Supporting information


Table S1.

## Data Availability

The data underlying this article are available in the Surveillance, Epidemiology, and End Results (SEER) database, at https://seer.cancer.gov/.
