# Supplementary material for: Caucasian validation of downstaging from IIB to IIA in T1N1M0 patients within the 9th edition of the non‐small cell lung cancer tumor‐node‐metastasis staging
Source: Cancer Med. 2024 Jul 24;13(14):e70018. doi: 10.1002/cam4.70018 (PMC11267448; doi:10.1002/cam4.70018)
Supplement: Supplementary file 1 — Table S1. [file CAM4-13-e70018-s001.docx]

**Table S1. Baseline characteristic comparisons in the IIA & IIA New matched pair and IIB & IIA New matched pair**

| Characteristic | IIA & IIA New matched pair | | | IIB & IIA New matched pair | | |
| --- | --- | --- | --- | --- | --- | --- |
|  | IIA (N=516) | IA New (N=516) | SMD | IIB | IIA New | SMD |
| Age, years |  |  | 0.0127 |  |  | -0.0175 |
| Continue (median, IQR) | 70 (64-76) | 70 (64-75) |  | 69 (64-74) | 69 (64-74) |  |
| Sex |  |  | 0.0699 |  |  | 0.0051 |
| Male | 259 (50.2) | 275 (53.3) |  | 316 (49.8) | 326 (50.2) |  |
| Female | 257 (49.8) | 241 (46.7) |  | 319 (50.2) | 323 (49.8) |  |
| Race |  |  | -0.0640 |  |  | 0.0225 |
| Caucasian | 491 (95.2) | 480 (93.0) |  | 604 (94.1) | 607 (94.5) |  |
| African | 15 (2.9) | 19 (3.7) |  | 29 (4.5) | 25 (3.9) |  |
| Other | 10 (1.9) | 17 (3.3) |  | 9 (1.4) | 10 (1.6) |  |
| Marital status |  |  | 0.0451 |  |  | 0.0841 |
| Married | 310 (60.1) | 280 (54.3) |  | 354 (55.1) | 353 (55.0) |  |
| Other | 206 (39.9) | 236 (45.7) |  | 288 (44.9) | 289 (45.0) |  |
| Insurance |  |  | 0.0194 |  |  | 0.0156 |
| Insured | 457 (88.6) | 467 (90.5) |  | 579 (90.2) | 576 (89.7) |  |
| Other | 59 (11.44) | 49 (9.5) |  | 63 (9.8) | 66 (10.3) |  |
| Tumor location |  |  | 0.0742 |  |  | -0.0326 |
| RUL | 333 (64.5) | 345 (66.9) |  | 402 (62.6) | 401 (62.5) |  |
| RML | 18 (3.5) | 16 (3.1) |  | 9 (1.4) | 9 (1.4) |  |
| RLL | 161 (31.2) | 155 (30.0) |  | 229 (35.7) | 230 (35.8) |  |
| LUL | 2 (0.4) | 0 (0.0) |  | 1 (0.2) | 1 (0.2) |  |
| LLL | 2 (0.4) | 0 (0.0) |  | 1 (0.2) | 1 (0.2) |  |
| Surgical extent |  |  | 0.0299 |  |  | 0.0730 |
| Lobectomy | 483 (93.6) | 485 (94.0) |  | 621 (96.7) | 623 (97.0) |  |
| Sub-lobectomy | 29 (5.6) | 26 (5.0) |  | 5 (0.8) | 3 (0.5) |  |
| Pneumonectomy | 4 (0.8) | 5 (1.0) |  | 16 (2.5) | 16 (2.5) |  |
| Chemotherapy |  |  | 0.0000 |  |  | 0.0000 |
| Not performed | 324 (62.8) | 324 (62.8) |  | 309 (48.1) | 309 (48.1) |  |
| Performed | 192 (37.2) | 192 (37.2) |  | 333 (51.9) | 333 (51.9) |  |
| Radiotherapy |  |  | -0.0157 |  |  | 0.0395 |
| Not performed | 495 (95.9) | 492 (95.3) |  | 629 (98.0) | 631 (98.3) |  |
| Performed | 21 (4.1) | 24 (4.7) |  | 13 (2.0) | 11 (1.7) |  |
| Histology |  |  | -0.0246 |  |  | 0.0388 |
| Adenocarcinoma | 276 (53.5) | 285 (55.2) |  | 343 (53.4) | 346 (53.9) |  |
| Squamous cell carcinoma | 151 (29.3) | 128 (24.8) |  | 199 (31.0) | 192 (29.9) |  |
| Other | 89 (17.2) | 103 (20.0) |  | 100 (15.6) | 104 (16.2) |  |
| Grade |  |  | 0.0615 |  |  | 0.0112 |
| Well | 48 (9.3) | 52 (10.1) |  | 27 (4.2) | 28 (4.4) |  |
| Moderately | 237 (45.9) | 236 (45.7) |  | 329 (51.2) | 329 (51.2) |  |
| Poor | 204 (39.5) | 201 (39.0) |  | 272 (42.4) | 271 (42.2) |  |
| undifferentiated | 4 (0.8) | 8 (1.6) |  | 3 (0.5) | 2 (0.3) |  |
| Unknown | 23 (4.5) | 19 (3.7) |  | 11 (1.7) | 12 (1.9) |  |
| VPI |  |  | 0.0000 |  |  | 0.0000 |
| Without | 516 (100.0) | 516 (100.0) |  | 642 (100.0) | 642 (100.0) |  |
| With | 0 (0.0) | 0 (0.0) |  | 0 (0.0) | 0 (0.0) |  |

IQR, interquartile range; RUL, right upper lobe; RML, right middle lobe; RLL, right low lobe; LUL, left upper lobe; LLL, left low lobe; VPI, visceral pleural invasion; SMD, standardized mean difference
